# Costing Health Benefit Packages Using the WHO UHC Compendium: A Proof-of-Concept Study in Kyrgyzstan

**DOI:** 10.34172/ijhpm.9333

**Published:** 2026-05-05

**Authors:** Gerard Joseph Abou Jaoude, Charlie Nederpelt, Saltanat Zhetibaeva, Gavin Surgey, Parisa Kamali Sadeghian, Neha Misra, Eliza Aidyldaeva, Mirlan Atakulov, Edil Orozaliev, Sven Neelsen, Christel Vermeersch, Baktygul Kambaralieva, Jolene Skordis, Tom Palmer, Rob Baltussen, Baktygul Isaeva, Hassan Haghparast-Bidgoli

**Affiliations:** ^1^Institute for Global Health, University College London, London, UK.; ^2^Radboud University Medical Center, Nijmegen, The Netherlands.; ^3^Ministry of Health, Bishkek, Kyrgyzstan.; ^4^World Health Organization, Geneva, Switzerland.; ^5^Mandatory Health Insurance Fund, Bishkek, Kyrgyzstan.; ^6^World Bank, Washington, DC, USA.; ^7^Japan International Cooperation Agency Kyrgyz Republic Office, Bishkek, Kyrgyzstan.

**Keywords:** Benefit Package, Costing, Priority Setting, Universal Health Coverage

## Abstract

**Background:**

Many countries are defining health benefits packages to progress toward universal health coverage (UHC). Cost estimates are required to ensure packages are affordable and implementable. However, a gap persists between global costing tools and recommendations informing package design and the way services are defined for country-level implementation. To address this, we developed the first health systems-wide costing tool and approach based on the World Health Organization (WHO) UHC Compendium database, which provides structured service definitions and data to facilitate country-level contextualisation and implementation. This paper presents our tool and approach through a proof-of-concept study in Kyrgyzstan.

**Methods:**

We developed a tool in Microsoft Excel that estimates normative economic costs for over 500 UHC Compendium services using a combination of bottom-up and top-down approaches. Resource use was derived from UHC Compendium metadata, supplemented with publicly available and country-specific data sources to inform input prices and population in need estimates. In Kyrgyzstan, all secondary data were validated and contextualised with national experts. We produced high-level cost estimates for 424 services identified as relevant for Kyrgyzstan and refined estimates for 181 priority services selected by country stakeholders. Sensitivity analyses investigated variations in personnel, medicine and overhead costs and currency fluctuations.

**Results:**

Delivering all 424 services at current utilisation levels, or low levels of utilisation for services not yet implemented, would cost an estimated US$ 186.97 per capita annually. Providing the 181 priority services would cost US$ 74.41 per capita at current utilisation levels and US$ 189.44 per capita if scaled to full (100%) utilisation. Costs were driven primarily by personnel and medicines. Sensitivity analyses showed costs ranging from US$ 58.76 to 90.05 per capita.

**Conclusion:**

This study demonstrates the feasibility of using UHC Compendium data to generate country-specific, service-level cost estimates for benefits package design. Other countries can adapt our tool and approach to design affordable and implementable packages in support of UHC goals.

## Background

**Key Messages**

**Implications for policy makers**

- We developed a practical, replicable tool and approach to cost services and inform health benefits package design using the World Health Organization (WHO) universal health coverage (UHC) Compendium, which contains structured service definitions and data to support country implementation.
- Results from our costing tool and approach effectively supported the evidence-informed prioritisation of services for Kyrgyzstan’s health benefits package reform, demonstrating proof-of-concept.
- This study provides the first comprehensive set of service cost estimates for Kyrgyzstan’s health system, alongside a tool and approach that can be adapted to guide similar reforms in other countries.

**Implications for the public**

Universal health coverage (UHC) aims to ensure everyone can access services they need without financial hardship. Health benefits packages, which involve defining publicly subsidised services that people should be entitled to, are considered a critical step towards UHC. To define a feasible benefits package, policy-makers must know the cost of services to secure adequate funding, expand access, and protect people from financial hardship. Through a case study in Kyrgyzstan, we describe an approach and tool we developed to estimate service costs for benefits package design. Our tool and approach effectively informed policy-makers deciding on which services to include in a package and informed different decisions requiring varying levels of precision over time. This is the first study to develop and apply a health systems-wide costing approach and tool using a recent World Health Organization (WHO) database of over 500 services, which can help other countries design well-defined, feasible health benefits packages.

A central challenge in the global push for universal health coverage (UHC) is determining which health services should be publicly funded. In Kyrgyzstan, significant healthcare financing reforms are being implemented to accelerate progress towards UHC.^1^ As in many countries, a key component of these reforms is the definition of a health benefits package, a set of health services provided free or partially subsidised for people at the point of use.^2,3^

 When designed through evidence-based and inclusive priority-setting processes, such packages can improve health outcomes, promote equity and protect people from financial hardship due to the costs of seeking care.^4^ Evidence-informed deliberative processes, used in Kyrgyzstan and other settings,^5,6^ guide stakeholders in identifying services, setting criteria and prioritising health services to include in an explicit benefit package. Service costs and budget impact are critical considerations for ensuring financial feasibility and sustainability.

 A growing body of evidence has emerged on the cost of globally recommended health services to inform priority setting, albeit with geographic disparities and a lack of country-level evidence for many services such as emergency care and the management of non-communicable conditions.^7-9^ Resources such as the Disease Control Priorities 3^rd^ Edition (DCP3),^10^ the One Health Tool,^11^ and World Health Organization (WHO)-CHOICE,^12^ provide cost estimates for health services that are more likely to be cost-effective in low- and middle-income settings.

 A major limitation of cost estimates based on global recommendations such as DCP3 is the gap between these global recommendations, associated cost data and the way health services are defined and delivered within national benefits packages.^13^ For example, DCP3 recommendations are often not integrated across care pathways and lack detail on required staff, medicines, diagnostics and other resources, making local adaptation challenging.^7,8,13^ Substantial additional work is typically needed to translate global guidance into implementable, country-specific health service definitions and cost estimates.

 To address this gap, this paper presents a costing tool and approach that we developed based on the WHO UHC Compendium database and its planning interface known as the Service Planning, Delivery and Implementation (SPDI) Platform. The costing tool we have developed operationalises SPDI structured service definitions and metadata to generate country-specific cost estimates. We demonstrate its feasibility and usefulness through a proof-of-concept case study in Kyrgyzstan, undertaken within ongoing health benefits package reforms in the country.

###  The Universal Health Coverage Compendium and SPDI Architecture

 WHO has developed the UHC Compendium database along with its planning interface known as the SPDI Platform.^13-15^ SPDI forms an integral part of the WHO UHC Toolkit of planning and costing tools, serving as a global resource to help countries design, implement, and monitor priority service packages for UHC. SPDI facilitates a structured approach to understanding current service delivery, provides an interface to support UHC package design and adds data on health workforce, health products, service delivery platforms and burden of disease to support integrated planning and implementation.

 SPDI is organised with a structured and nested architecture that is aligned with WHO guidelines and expert consensus,^13-15^ based on the WHO family of international classification systems such as the International Classification of Diseases (ICD-11). The SPDI hierarchical architecture includes over 500 bundles of health services, each representing a strategic “choice point” for countries designing a benefits package. Bundles are linked to detailed metadata for planning, delivery and costing and can be combined to reflect care pathways, such as screening and treatment for hypertension. A bundle of health services consists of one or more standardized visits, which serve as operational units of contact within the health system, comprising one or more actions (eg, history and physical examination, or prescribing a medicine) that are promotive, preventive, diagnostic, therapeutic, resuscitative, rehabilitative, or palliative services delivered to defined target populations.^15^ Visits represent typical care encounters, and nine default visit types are included:

- Brief visit
- Expanded visit
- Comprehensive visit
- Basic emergency unit visit
- Advanced emergency unit visit
- Hospital stay (numbers of days)
  ♦ Basic theatre-based procedure
  ♦ Advanced theatre-based procedure
  ♦ Critical care (numbers of days)

For every visit, the additional metadata specifies:

- The proportion of the target population expected to require the action,
- The health worker type needed (aligned with ISCO-08 classifications),
- The estimated time to deliver each task, and
- Associated health products (medicines, diagnostics, medical devices, and consumables) mapped to WHO normative references such as the Essential Medicines List, Essential Diagnostics List, and the Priority Medical Devices Information System (MeDevIS) database.

**Figure 1:**
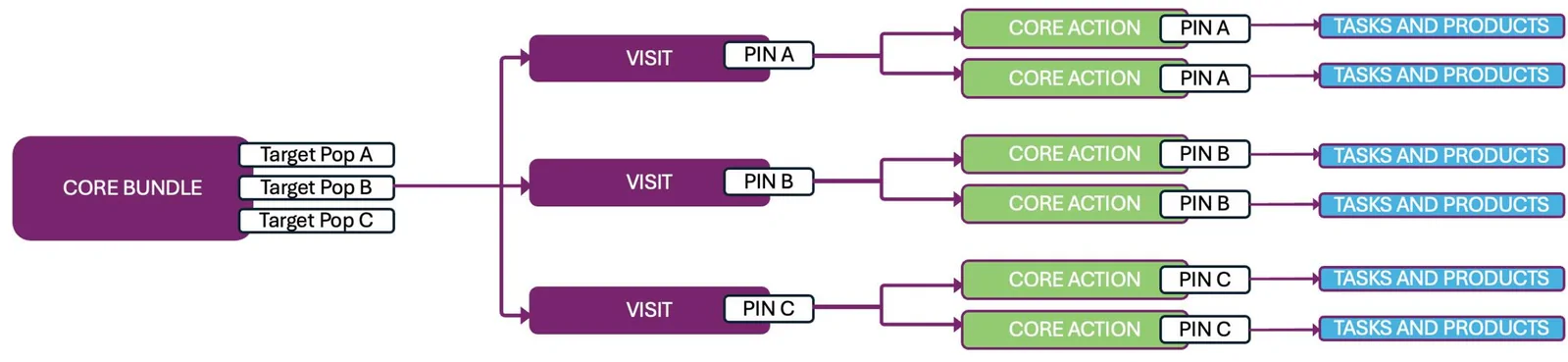
WHO SPDI Architecture That the Costing Tool Is Based on. Abbreviations: PIN, Population in need; WHO, World Health Organization; SPDI, Service Planning, Delivery and Implementation. Source: Correspondence with WHO. Note: Bundles represent groups of services or actions. Core actions are those intrinsic to a given bundle, while additional actions are extended services that may not be prioritized in all settings.

Figure 1 presents the SPDI architecture and structure of a bundle, which is illustrative of the full bundle dataset included in Supplementary file 1. This structured approach supports detailed resource mapping and facilitates country-level adaptation. For example, in diabetes care, different patient subgroups can be assigned distinct bundles: some requiring insulin-based regimens, others oral medications such as metformin, and others periodic monitoring and counselling. Through its flexible digital interface, SPDI allows policy-makers to tailor service definitions and associated resource inputs to their specific delivery capacity and local priorities, while maintaining alignment with global standards, and export data to support evidence-informed package design and implementation planning.^15^

 Despite the strengths of the SPDI platform, no costing model had previously been developed using its structured service definitions and data. Developing such a model based on SPDI offers a unique opportunity to support the design of well-defined, implementable benefits packages by generating country-specific cost evidence to inform policy decisions. Next, we introduce the context in Kyrgyzstan, within which we developed and applied our costing tool and approach to estimate the costs of 424 bundles of health services defined using SPDI as part of ongoing national health benefits package reforms.

###  Case Study Context: Health Financing and Service Delivery in Kyrgyzstan

 Kyrgyzstan spends significantly less on health than comparable Central Asian and European countries.^1,16^ In 2022, national health spending was just US$ 86 per capita, representing only 4.9% of gross domestic product, of which US$ 40 was accounted for by general government health expenditure.^17^ The country currently operates a mandatory health insurance system that covers roughly 69% of the population.^16^

 The Mandatory Health Insurance Fund (MHIF) pools contributions and budget transfers to purchase services defined in the State-Guaranteed Benefits Package (SGBP), which covers outpatient and inpatient care delivered through a tiered system comprising:

1. *Feldsher-obstetric points*: established to improve access to primary care in rural areas, staffed by a feldsher (practitioner without full qualifications) and a family doctor that regularly visits.
2. *Family group practices*: commonly the first point of contact with the health system, staffed by family doctors providing primary care.
3. *Family medicine centres*: more specialised staff that care for patients referred by family doctors.
4. *District hospitals*: first-level referral hospitals.
5. *Regional and tertiary hospitals*: second-level and third-level referral hospitals.

While domestic public funds account for 46% of national health spending, levels of private out-of-pocket spending remain high at 38%.^17^ These costs, mainly from medication co-payments and gaps in insurance coverage, lead to financial hardship for over 1 in 10 households using health services, disproportionately affecting the poorest quintiles.^1,16^ Meanwhile, the health system faces rising demand due to a double burden of persistent infectious diseases and growing rates of non-communicable diseases, which have become the leading cause of mortality.^1,16^

 In response, the government launched reforms to revise the SGBP with the goal of expanding coverage and strengthening equity. This study is part of a wider World Bank-funded project supporting these reforms through an evidence-informed deliberative process.^5,6^ Technical Working Groups (TWGs), including national experts and health system stakeholders, reviewed data on the SPDI platform and adapted 424 bundles of health services to the Kyrgyzstan context. Through a structured prioritization exercise, guided by the steps outlined in evidence-informed deliberative process methodology,^5,6^ 181 bundles of health services were identified to be of highest priority and are under consideration for inclusion in the revised benefits package. In brief, TWGs and policy-makers agreed on eight decision criteria^1^ to guide priority setting and evidence was generated based on decision criteria for each of the 424 services considered. Evidence on decision criteria was systematically appraised for each service by TWGs through structured deliberations, aimed at reaching consensus across group members, which resulted in the recommendation to prioritise 181 services for policy-makers to approve. More details on the prioritisation process and final results from the wider project will be published elsewhere.

 This paper focuses on the costing tool and approach that we developed and applied within the SGBP revision process to inform priority setting. Below, we outline the methods underpinning the costing tool we developed using UHC Compendium data from SPDI, the approach employed to cost services using the tool in Kyrgyzstan and how cost estimates informed benefits package design. We then report case study results for Kyrgyzstan to illustrate feasibility and policy relevance, before discussing generalisability to other settings. Our experience offers an approach and costing model that other countries can adapt to support evidence-informed priority setting and define sustainable, implementable health benefits packages.

## Methods

###  Costing Tool Overview

 Using UHC Compendium data exported from the SPDI Platform in April 2024, we developed a Microsoft Excel–based costing tool drawing on the data sources described in Table. The tool combines bottom-up (personnel, medicines, and diagnostics) and top-down (consumables and overheads) costing approaches to estimate the normative economic cost of over 500 bundles of health services contained within SPDI and the underlying UHC Compendium database.^18,19^ Details on the calculations carried out within the tool are included in Supplementary file 2. The tool was developed to be flexible and easily adaptable to varying policy-maker needs, timelines and data availability, providing options for rapid and more detailed costing approaches (Supplementary file 1). Next, we describe the two stages of costing carried out to inform benefits package reforms in Kyrgyzstan using the tool, before explaining our costing approach and how the tool was applied in practice.

**Table:** Summary of Costing Tool Variables and Corresponding Sources for Pre-loaded and Kyrgyzstan Analysis Data

**Variable Category**	**Variable**	**Pre-Loaded Costing Tool Data Source**	**Data Source Used in Kyrgyzstan**
Personnel	Types of outpatient visits and hospital stay	UHCC	TWGs and UHCC
	Annual frequency of outpatient visits and hospital stay	UHCC	TWGs, protocols, MHIF, and UHCC
	Medical procedure length in minutes	UHCC and authors’ classification	TWGs and UHCC
	Average cost of outpatient visits and hospital stay	Time-based costing using wages from Serje et al and WHO-CHOICE^20-22^	WHO-CHOICE^20,21^
	Health worker salaries	Serje et al and WHO-CHOICE^20-22^	National salary registers^23,24^
Medicines	Medicine name, dosage, route of administration and unit	UHCC	TWGs and UHCC
	Number of medicine units required per day	UHCC	TWGs and UHCC
	Annual treatment duration in days	UHCC	TWGs and UHCC
	Average cost per medicine unit	(1) WHO Global Price Reporting Mechanism,^25^ (2) International Medical Products Price Guide,^26^ (3) Published literature, (4) Dutch national price reporting mechanism^27^	Kyrgyzstan and Uzbekistan national medicines price lists^28,29^
Diagnostics	Name of diagnostic or laboratory test	UHCC	TWGs and UHCC
	Type of diagnostic/laboratory test (point of care, basic or advanced)	UHCC	UHCC
	Average cost by type of diagnostic/laboratory test	(1) International Medical Products Price Guide,^26^ (2) OneHealth Tool,^11^ (3) Literature review, (4) Category averages from literature review^30,31^	Literature review^30,31^
Products and consumables	Name of reusable medical device and a “Pack number” linked to the WHO MeDevIS	UHCC	Not used, percentage mark-up instead
	Percentage mark-up for products and consumables	Approximation of proportions reported in LMIC published literature	PHC capitation and diagnosis-related group costing studies^32,33^
Facility-level capital and overheads	Name of Capital medical device and ‘Pack number’ linked to the WHO MeDevIS	UHCC	Not used, percentage mark-up instead
	Percentage mark-up for capital and overheads	Approximation of proportions reported in LMIC published literature	PHC capitation and diagnosis-related group costing studies^32,33^
Population in need	Defined population in need of services or actions in a visit	UHCC	TWGs and UHCC
	Number of people in need of services or actions in a visit	Multiple sources	Multiple sources
	Percentage of the service-level population in need of a health visit	UHCC	UHCC
	Percentage of the visit-level population in need of various health services performed during a visit	UHCC	UHCC
Service utilisation	Percentage of people utilising a service at each service delivery level	UHCC	TWGs and UHCC
	Overall percentage of population in need utilising a service	Multiple sources	Multiple sources

Abbreviations: WHO, World Health Organization; TWGs, Technical Working Groups; MHIF, Mandatory Health Insurance Fund; LMIC, low- and middle-income country; UHCC, Universal Health Coverage Compendium; MeDevIS, Medical Devices Information System; PHC, primary healthcare.

###  Two-Stage Costing in Kyrgyzstan 

 To maximise the validity and policy relevance of our cost analysis, we engaged extensively with TWG experts and other key stakeholders, resulting in a two-stage costing approach. This involved a first stage of data collation and review to generate “high-level” cost estimates for all 424 service bundles considered, followed by a second stage to generate “refined” cost estimates for the 181 prioritised service bundles. To support systematic data collation and ensure a consistent review process, we developed standardized data collection templates in Microsoft Excel (Supplementary file 1). Several training workshops were held with TWG members to explain the underlying model assumptions and guide consistent use of the templates across all groups.

 In the *first stage*, TWGs reviewed the content and definitions of bundles of health services structured through the SPDI Platform and adapted them to the Kyrgyzstan context. This included revising parameters such as the number and type of visits, medicines, and the defined populations in need. These adaptations informed high-level cost estimates for all 424 bundles initially considered for inclusion in the benefits package. The estimated cost of delivering the full set of bundles far exceeded available fiscal space. Alongside other decision criteria, such as cost-effectiveness and burden of disease, these results informed evidence-based deliberations among TWGs and policy-makers, leading to the prioritisation of 181 bundles of health services identified as most critical.

 In the *second stage*, additional data collection and review were undertaken to refine the cost estimates for these 181 priority bundles of health services. This phase primarily focused on working with TWGs to contextualise unit costs for medicines, high-cost consumables and to update population-in-need estimates based on the best available local data. The resulting refined estimates are currently being used to inform the final round of prioritisation to determine which bundles will be included in the health benefits package for implementation.

 A timeline of this costing study, with key milestones, is included in Supplementary file 3. Over a six-month timeframe, both stages of data collection and validation were completed. By the second stage, almost all input data were reviewed, adjusted, and where necessary, replaced to ensure relevance to the Kyrgyzstan context. As noted in Table, TWGs systematically amended default SPDI parameters to improve alignment with local practices and delivery models to reflect the Kyrgyzstan context. In the next section, we describe how these variables and cost data were integrated into the costing tool developed and applied in Kyrgyzstan.

###  Costing Approach and Tool Application in Kyrgyzstan

 Costs were estimated from the provider perspective (ie, Ministry of Health and MHIF) over a 12-month time horizon. Our costing approach was guided by principles outlined in the Global Health Costing Consortium reference case,^18^ developed as a gold standard for costing health interventions in low- and middle-income countries and used by previous studies costing health benefits packages.^34^ The base year of analysis was 2023 and all prices were captured in their original currency and price year, primarily Kyrgyz Som. Prices were then inflated to 2023 values before conversion to US dollars, respectively using World Bank GDP deflator data and official average annual exchange rates (Som 87.86 per US$ 1 for 2023).^35^ Below, we describe how each cost component was estimated using the tool in Kyrgyzstan.

####  Personnel Costs

 Personnel costs were estimated separately in the costing tool for: (1) outpatient visits and hospital stays, and (2) medical procedures such as haemodialysis, surgeries or biopsies.

 We estimated personnel costs per type of outpatient visit and hospital stay by drawing on the WHO-CHOICE database.^20,21^ The “Comprehensive visit” was used as the reference outpatient cost and “Hospital stay” was used as the reference inpatient cost. Scaling factors were then applied to adjust these reference values to reflect the full range of visit types defined in the SPDI Platform, based on expert and TWG assumptions on the personnel time required for different visit types (Supplementary file 3). Service delivery levels comprised of: (1) feldsher and family group practices, (2) family medicine centres, (3) first-level referral hospitals, and (4) second-level and third-level referral hospitals. The resulting costs per visit type and delivery level were multiplied by the estimated annual frequency of visits or length of stay to calculate total annual costs.

 In addition to visits and hospital stays, the SPDI Platform includes medical procedures and surgeries such as “Mastectomy” or “External haemorrhage control with tourniquet application.” Personnel costs for these procedures were estimated separately from a facility-based visit or hospital stay. To estimate the personnel costs per medical procedure or surgery, we employed an ingredients approach^19^ by multiplying the length of a procedure in minutes with the corresponding salary cost per minute of health workers required to carry out the procedure. Official annual salaries of health personnel in Kyrgyzstan^23,24^ were converted to costs per minute assuming 40 hour working weeks and 85% productive time.^36,37^

####  Medicines

 Medicine costs were estimated using the Defined Daily Dose methodology and an ingredients approach in the tool.^19^ This was done by standardising medicine costs to a cost per unit based on the specified dosage, which was then multiplied by the number of units per day and annual treatment duration in days. Medicine costs were based on the Kyrgyzstan national medicines price list,^28^ which reported average annual prices paid per medicine. Where Kyrgyzstan national medicines price data was unavailable, we used the Uzbekistan national medicines price list.^29^ All medicine unit costs are included in Supplementary file 4.

####  Diagnostics

 To meet crucial policy timeframes, we did not cost each diagnostic test using an ingredients approach in the tool, especially when considering that the budget impact of packages is typically driven by staff and medicines costs. In the SPDI platform, diagnostic tests are categorised into three categories (1) point of care tests, (2) basic laboratory tests, and (3) advanced laboratory tests, which include genomic testing where indicated. We therefore estimated diagnostic costs based on these three categories, and a separate consideration of genomic testing within advanced laboratory tests, rather than costing each individual test listed. The average cost per diagnostic testing category was estimated through an opportunistic literature review^30,31^ restricted to studies with data on low- and middle-income countries, which was then reviewed and validated by experts. Diagnostics costs can be found in Supplementary file 3.

####  Consumables and Facility-Level Overheads

 To estimate the cost of consumables and facility-level overheads in the tool, we applied percentages to the summed cost of personnel, medicines, and diagnostic costs (Supplementary file 3). Different consumable and facility-level overhead percentages were applied based on service delivery levels. The percentages were informed by mean facility-level expenditure estimates sourced from the Kyrgyzstan primary healthcare (PHC) capitation costing study (for feldsher, family group practices, and family medicine centres)^32^ and the diagnosis-related groups costing study (for first-level, second-level and third-level referral hospitals).^33^ Mean percentages for facility-level overheads, per service delivery level, captured utilities, maintenance and repairs, administration, equipment, vehicles, operating expenses, cleaning and sanitation, among other indirect or joint costs applicable to a given service delivery level. High-cost consumables packs, such as for dialysis, were costed separately using an ingredients approach.

####  Estimating Budget Impact

 Budget impact was estimated in the tool based on the population in need for a given service and the percentage of the population in need utilising the service. Country-level utilisation estimates per service were sourced from a range of established global sources, including WHO and the United Nations agency databases, with TWG and DCP3 assumptions used for services without available data. At the level of an outpatient visit or hospital stay, which comprises a set of health actions within a given health service, over 600 populations in need were estimated based on publicly available data (Supplementary file 4). We drew on a range of established global sources, which include population size and burden of disease estimates for Kyrgyzstan, alongside a comprehensive review of the literature. We considered the following criteria when selecting data on which to base our population in need estimates:

1. *Location*: Kyrgyzstan estimates were prioritised, followed by neighbouring countries and Central Asia regional estimates, followed by other regional and global estimates.
2. *Geographic scope*: National estimates were prioritised over sub-national estimates.
3. *Modelled estimates*: Programme-specific modelled estimates for conditions such as HIV or diabetes (eg, the Joint United Nations Programme on HIV/AIDS, International Diabetes Federation) were prioritised over sector-wide modelled estimates such as the Global Burden of Disease study by the Institute for Health Metrics and Evaluation.^38^
4. *Reviews and meta-analysis*: Pooled estimates from meta-analyses and systematic reviews were prioritised over single study estimates.

The relationship between costs and service utilisation at scale was assumed to be linear in the tool. In other words, the average cost per service user was assumed to remain constant as the number of people in need using a service increases. This choice was made given the absence of data to inform otherwise, the number of services costed, existing levels of service coverage and the single year time horizon considered.^18^ When estimating budget impact, expert assumptions on the percentage of people accessing care across different health service delivery levels were used to disaggregate populations in need at the health service delivery level. The latter were then multiplied by the average cost per service user at a given service delivery level to estimate the annual total cost of implementing a given service. An equivalent annual cost per capita of implementing a given service was then estimated by dividing the annual total cost of implementing a service by the total population in Kyrgyzstan (n = 6 839 606) for the year 2023.^39^

####  Sensitivity Analyses

 We carried out a two-way sensitivity analysis to assess the impact of a 25% relative increase or decrease of key cost drivers for the prioritised subset of 181 bundles of health services, namely by varying personnel and medicines costs. Relative variations of 25% were deemed reflective of potential changes to national salaries for health personnel and the largest likely variations in medicine costs. We also carried out a univariate sensitivity analysis to assess the impact on results of a 50% relative increase or decrease in the percentage mark-up for overheads. This was to reflect uncertainty based on variations in the percentage mark-up reported for overheads across facilities sampled in the PHC capitation and diagnosis-related group costing studies.^32,33^ The potential impact of currency fluctuations was assessed for non-personnel costs given that salaries are paid for in local currency. This involved a univariate sensitivity analysis investigating the impact of a conservative 10% relative increase or decrease in the Kyrgyz Som to US$ exchange rate based on central bank data,^40^ which reported maximum currency fluctuations of around 5%.

## Results

**Figure 2:**
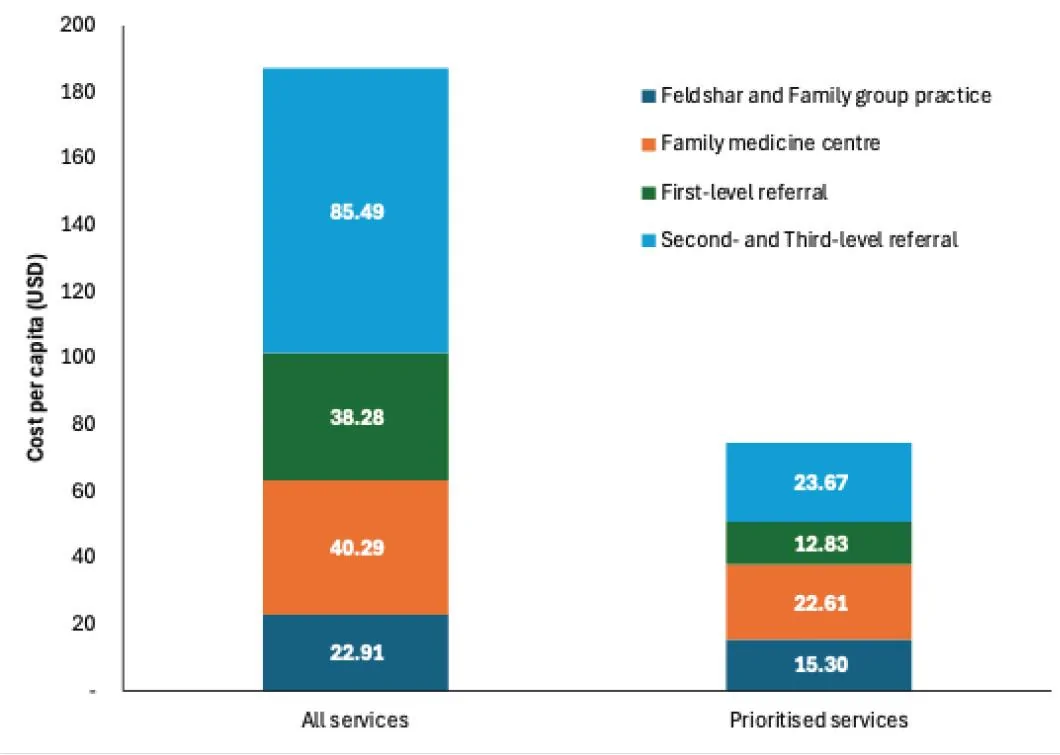
Cost Per Capita of All Services Considered and Prioritised by Service Delivery Level (2023 US$).

 The estimated cost to providers of delivering all 424 bundles of health services considered during the initial prioritisation was US$ 186.97 per capita at current levels of utilisation for bundles already implemented, or assuming 25% utilisation among populations in need of bundles not yet provided (Figure 2). PHC delivery accounted for 33.8% (US$ 63.20 per capita) of these total costs, including US$ 22.91 per capita for bundles delivered through feldsher and family group practices and US$ 40.29 per capita through family medicine centres.

 Following an evidence-based prioritisation, 181 bundles of health services were identified as the highest priority for inclusion in the benefits package. All prioritised bundles were already implemented to some extent in the country. First stage “high-level” cost estimates for the 181 bundles were 15.56% lower overall compared with the second stage “refined” estimates that we present here and which informed final prioritisation decisions. The estimated cost to deliver the 181 prioritised bundles at current utilisation levels was US$ 74.41 per capita (Figure 2). Within this estimate, PHC accounted for 51.0% of costs, including US$ 15.30 per capita (20.6%) through feldsher and family group practices and US$ 22.61 per capita (30.4%) through family medicine centres. Estimated costs for first-level referral hospitals were US$ 12.83 per capita (17.2%) and US$ 23.67 per capita (31.8%) for second- and third-level referral hospitals. Costs per capita for each of the 181 prioritised bundles are detailed in Supplementary file 5 and ranged from near zero up to US$ 5.56 per capita under existing levels of utilisation.

 Mean annual costs per service user varied substantially across services. Counselling services typically had the lowest costs, such as the prevention of chronic non-communicable diseases through integrated counselling on healthy diet, physical activity, weight management, and alcohol and tobacco use – which had a mean annual cost of US$ 4.23 per service user. Cancer services, dialysis and the management of complex conditions had the highest cost, such as the management of primary immunodeficiencies with surgical intervention – which had a mean annual cost of US$ 17 039.29 per service user.

**Figure 3:**
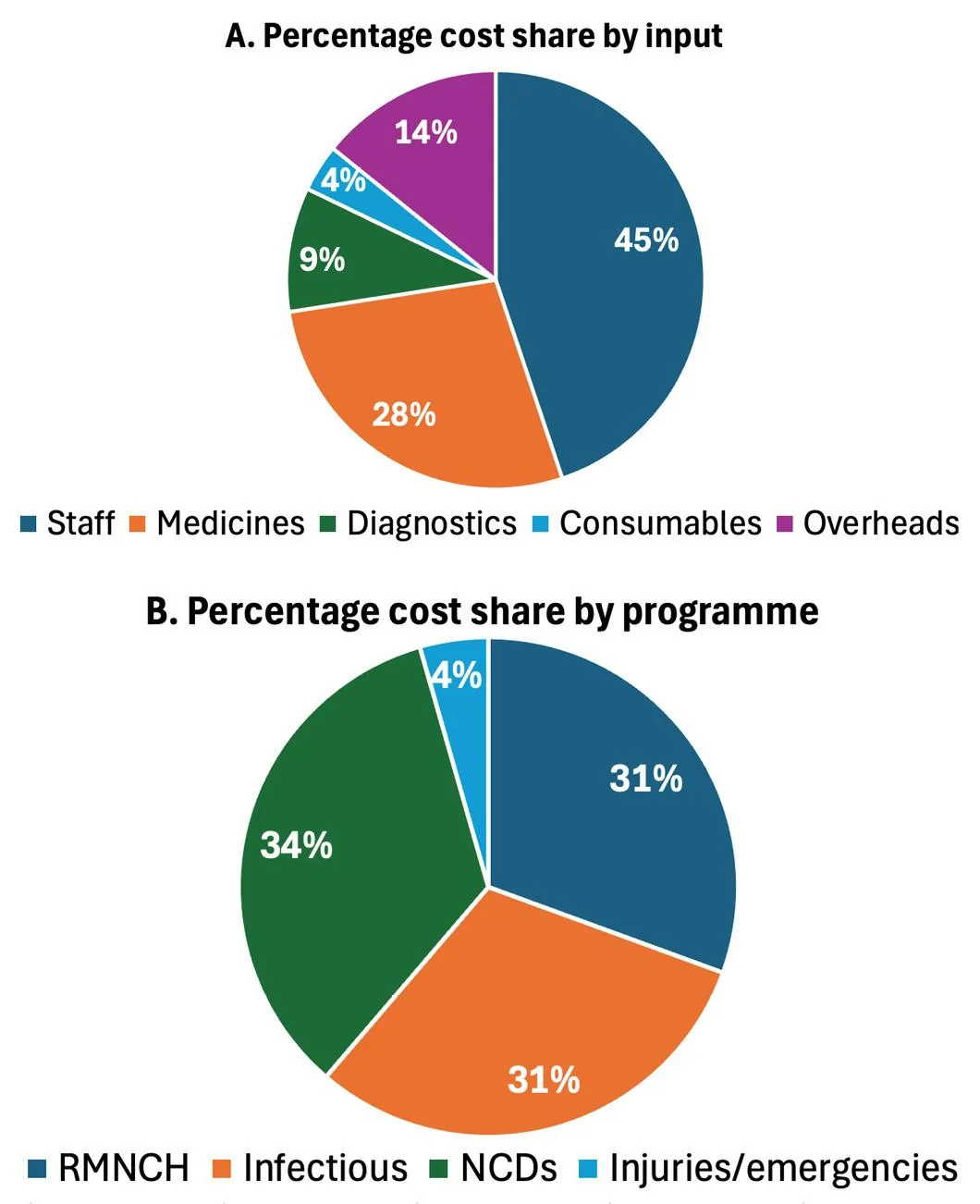
Percentage of the Estimated Cost of Prioritised Services by Input and Programme Area. Abbreviations: RMNCH, reproductive, maternal, neonatal and child health; NCDs, non-communicable diseases.

 Personnel and medicines were the main cost drivers, accounting for 45% and 28% of the total estimated costs for prioritised bundles, respectively (Figure 3A). The remaining 27% consisted of facility-level overheads (14%), diagnostics (9%), and consumables (4%). When analysed by programme area (Figure 3B), nearly all costs were distributed across reproductive, maternal, neonatal and child health (34%), infectious disease (31%), and non-communicable disease (31%) bundles. Only 4% of total costs were attributed to bundles related to injuries and emergencies.

**Figure 4:**
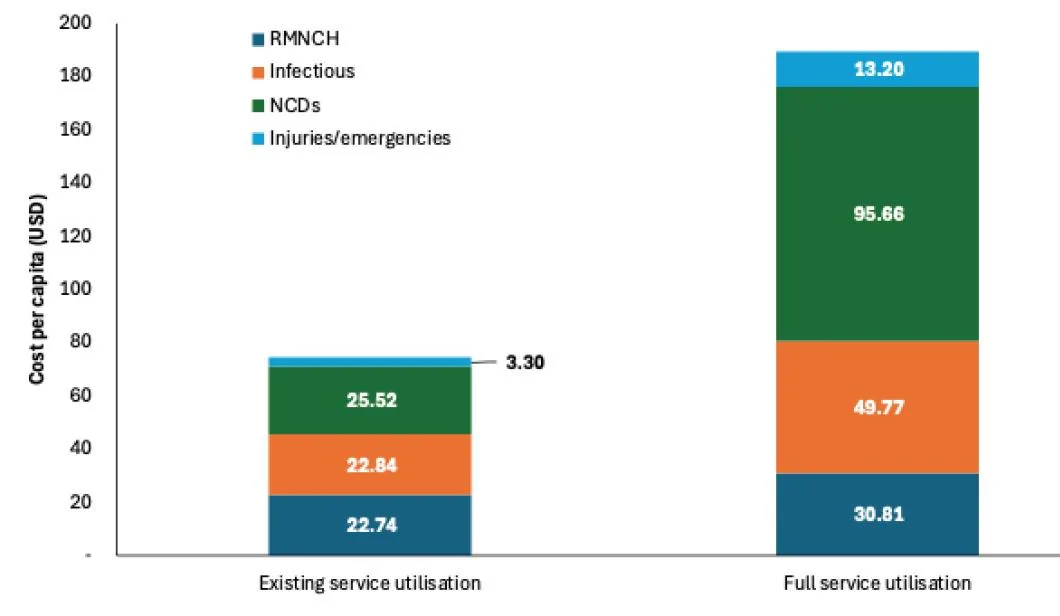
Cost Per Capita of Providing Prioritised Services at Existing and Full Levels of Service Utilisation, Disaggregated by Programme Area (2023 US$). Abbreviations: RMNCH, reproductive, maternal, neonatal and child health; NCDs, non-communicable diseases.

 If all 181 prioritised bundles of health services were delivered at full coverage (ie, 100% utilisation), the estimated cost would increase to US$ 189.44 per capita – an additional US$ 115.03 per capita compared with existing utilisation levels (Figure 4). The incremental costs of scaling up varied substantially by programme area. Full coverage of reproductive, maternal, neonatal and child health bundles would require an additional US$ 8.06 per capita (+35%), whereas scaling up infectious disease bundles would cost an additional US$ 26.92 per capita (+118%). Non-communicable disease bundles represented the largest incremental cost, at US$ 70.14 per capita (+275%), followed by injuries and emergency bundles, at US$ 9.90 per capita (+300%).

**Figure 5:**
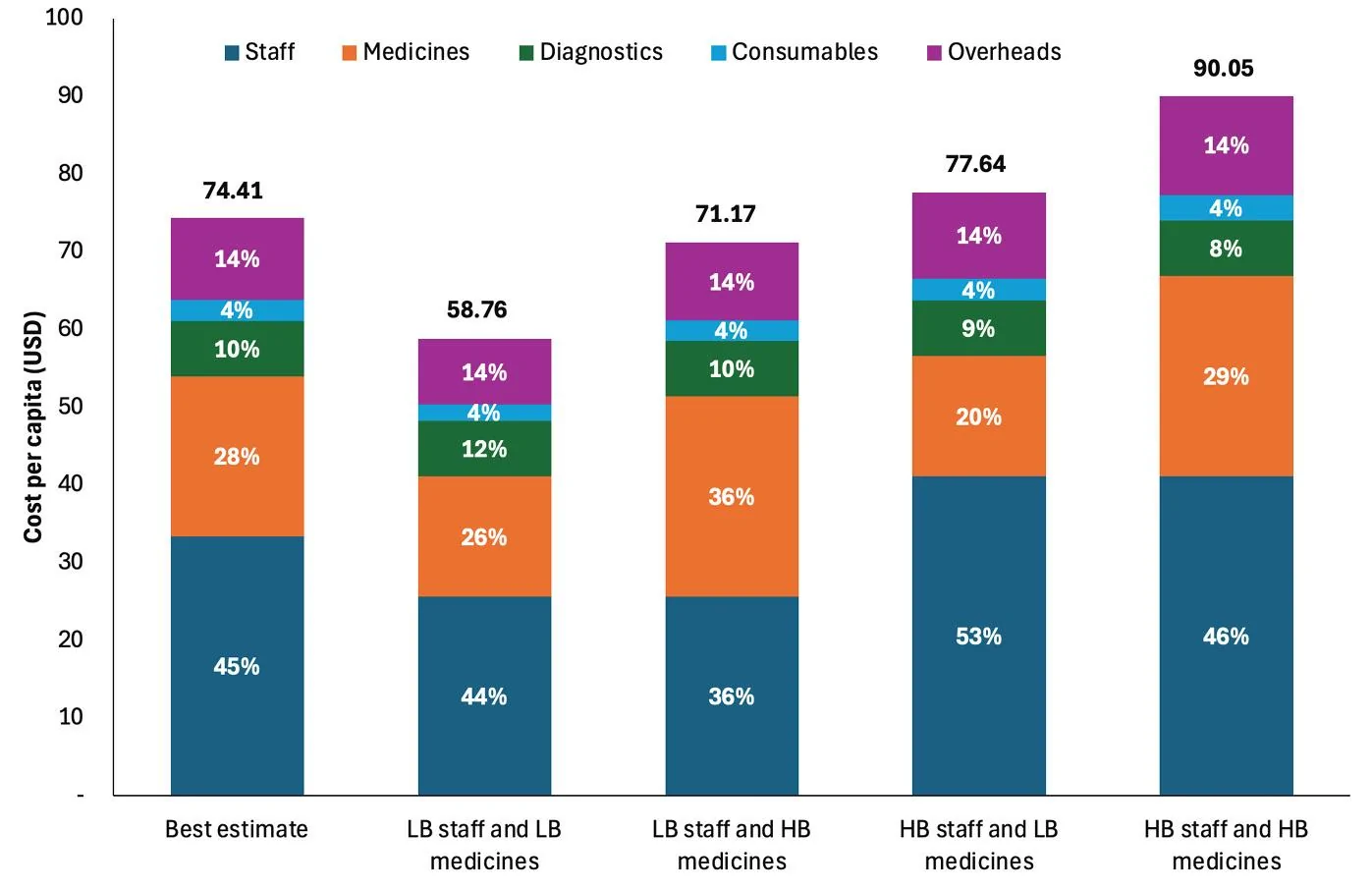
Two-Way Sensitivity Analysis Results With Percentage Cost Shares by Service Input and Total Cost Per Capita (2023 US$). Abbreviations: LB, low-bound, -25%; HB, high-bound, +25%.

 As described in the methods, a two-way sensitivity analysis assessed the impact of uncertainty in personnel and medicines costs. If both inputs were 25% higher than in the base case, the estimated cost of delivering the prioritised bundles at current utilisation would rise to US$ 90.05 per capita (Figure 5). If both inputs were 25% lower, the estimated cost would decrease to US$ 58.76 per capita. The total per capita cost therefore varied by ±US$ 15.65 compared to the main estimate of US$ 74.41 per capita. Univariate sensitivity analysis of a 50% relative decrease or increase in overheads costs results in respective estimated costs of US$ 69.12 and US$ 79.69. A 10% relative increase or decrease in the Kyrgyz Som to US$ exchange rate, applied to non-personnel cost components, results in respective estimated costs of US$ 70.68 and US$ 78.97.

## Discussion

 This paper presents the first development and application of a health systems-wide costing model based on UHC Compendium data from the WHO SPDI Platform, part of WHO’s UHC Toolkit designed to help countries define clear, implementable health benefits packages for UHC. We estimated that delivering the 181 prioritised bundles of health services in Kyrgyzstan would cost US$ 74.41 per capita at existing levels of utilisation. This exceeds current government health spending, underscoring the importance of aligning the ambitions of the benefits package with available fiscal space.

 The approach developed in this study produced timely results and informed critical decisions throughout the priority-setting process. Other countries using the SPDI platform to design or revise their benefits packages can adapt this costing model to fit their context, data availability, and policy timelines. A user interface, guidance materials and a public version of the model are under development and will be made freely available to support broader adoption.

 Recent reviews of studies costing health benefits packages document substantial heterogeneity in methods, findings and reporting, a lack of uncertainty analyses in some cases, and insufficient service-level data as a constraint in several studies, leading to calls for more standardisation of methods and closer links to budgeting and implementation.^41,42^ Our costing tool and approach align with several of these recommendations by leveraging the UHC Compendium database, which provides structured service definitions and input data that can be easily contextualised to local health systems for implementation. Our costing tool employs transparent, normative ingredients-based costing (used in three-quarters of reviewed studies^41^) for personnel, medicines, diagnostics, and high-cost consumables, complemented with explicit mark-ups for overheads and the reporting of deterministic sensitivity analyses. Nevertheless, our reliance on linear scale-up assumptions and the use of markups for overheads reflect commonly faced challenges when costing benefits packages.^41,42^

 The information needs of policy-makers often influence the level of accuracy required in cost estimates. This involves a trade-off between the desired accuracy and the time and resources available to produce these estimates.^19^ Ideally, estimates should be as detailed as necessary for the specific policy decisions they inform. In Kyrgyzstan, as in other countries redefining their benefits packages, initial high-level estimates were sufficient to shortlist and prioritise bundles from a long list of options.^43^ More refined costing for the prioritised bundles was then undertaken to compare against fiscal constraints, with further adjustments planned to support implementation and purchasing. The design of our approach and tool reflected this reality. Recognising data limitations in Kyrgyzstan and other low- and middle-income countries, the model is intentionally flexible, allowing users to tailor the level of detail in estimates based on available resources and timelines rather than requiring exhaustive data upfront.

 In practice, our model and approach supported key policy milestones without duplicating efforts. Initial cost estimates for the priority 181 bundles from a potential 424 bundles of health services were generated within three months, using largely pre-loaded, readily accessible secondary data. During the prioritisation process, these estimates were progressively refined by validating and contextualising data on medicines, high-cost consumables and populations in need. Moving forward, the estimates presented here can be further developed to inform implementation planning by incorporating national programme costs, more detailed data on equipment and capital investments, and micro-costing of diagnostics and consumables.

 Beyond informing package design, the analysis also highlighted important insights for broader health policy in Kyrgyzstan. For example, we found that around 45% of prioritised service costs are accounted for by personnel, in contrast to 60%-70% according to national budgets depending on the service delivery level.^44^ This does not indicate excessive numbers of health personnel or salaries, Kyrgyzstan has among the lowest rate of health workers in the WHO European Region.^1^ Rather, our estimated difference in cost shares is supportive of previous findings highlighting under-investment in medicines, diagnostics and effective monitoring of care in the public sector,^1,16^ particularly for non-communicable conditions. For example, while 49.0% of adults living with hypertension are on treatment, only 11.3% have controlled blood pressure.^45^ Similarly, only half of diabetes patients receive routine blood glucose monitoring, with risks to blood-glucose control.^46^ A key next step when implementing a final package of services in Kyrgyzstan, as in many other settings, will be to strengthen medication supplies and quality of care for non-communicable diseases.

 Overall, little data or evidence are available to compare against our findings. Personnel and medicine costs were the main cost drivers in our study, in line with previous benefits package costing studies reported on by previous reviews.^41,42^ At the country level, data are primarily related to spending on actual standards of care, while our findings are bottom-up normative cost estimates that are based on ideal standards of care. That said, as would be expected, our overall estimate that US$ 74.41 per capita is required to provide prioritised services at existing utilisation levels is higher than overall comparable national health spending in 2023^[1]^. It is more challenging to compare by programme area given that there is little recent spending data available at this level of disaggregation. It was nonetheless possible to compare our costs against spending estimates for tuberculosis and diabetes – both of which are key national health priorities.^1^ We estimated that tuberculosis services cost US$ 2.42 and diabetes services cost US$ 6.50 per capita, compared with respective spending estimates of US$ 2.70^47^ and US$ 8.66 per capita.^48^ In these cases, we expect spending to be higher than our estimates given that we did not capture national-level activities or any population and public health measures.

 Although our results show higher uncertainty in projected costs at full scale compared to current utilisation, they clearly demonstrate the substantial investment required to expand coverage. The smallest incremental costs were for maternal, neonatal, and child health bundles of services, reflecting progress in this area over the past two decades.^49^ By comparison, the largest additional costs at scale are required to provide services for non-communicable conditions, which are now the leading cause of death in Kyrgyzstan.^1^ Given the growing economy, low health spending relative to comparable countries and a reduction in the share of government spending allocated to health,^1^ these results underline the need for prioritising health and substantially increasing health funding to meet political commitments – particularly for non-communicable conditions.

 Several limitations should be considered when interpreting these results. This study represents the first development and application of a systems-wide UHC Compendium costing model and focuses on current costs rather than projections over time. As such, substantial uncertainty surrounds the estimates at scale, and future iterations could incorporate non-linear cost functions and model gradual increases in utilisation. In addition, service-level data on utilisation by delivery platform remain limited in Kyrgyzstan, as in many low- and middle-income settings. We addressed these gaps through structured expert consensus to replace or adapt default WHO UHC Toolkit data where needed. In the future, developing data-driven assumptions on utilisation by delivery platform by service type or programme area could further improve accuracy. It is also important to note that data and assumptions informing results presented in this study are localised to Kyrgyzstan and are not generalisable to other contexts. However, pre-loaded global data in the costing tool that we have developed can be contextualised to other setting using the approach we have set out. Finally, our cost estimates were designed to inform prioritisation, not operational planning, with broad assumptions on diagnostic costs and percentage markups for consumables and overheads – all of which may have affected the accuracy of our estimates. Nonetheless, our cost estimates do offer a robust basis for subsequent work to inform implementation.

## Conclusions

 An increasing number of countries are using the WHO’s UHC Toolkit to support the development of clear, implementable health benefits packages that advance progress toward UHC. This paper has presented the first flexible, health systems-wide tool and approach specifically designed to cost health services using UHC Compendium data from the SPDI platform. We developed, tested, and applied this costing tool in Kyrgyzstan, generating timely estimates that directly informed key policy decisions during benefits package reforms. While the analysis has some limitations, including data gaps and uncertainty around scaling assumptions, the model offers a practical, adaptable tool that can be used to inform priority setting, resource planning, and package design in other settings. As more countries work to operationalise UHC commitments, this approach provides a replicable example of how structured, transparent costing can help align policy ambitions with available resources.

## Acknowledgements

 A special thank you to John Fogarty and Teri Reynolds from the WHO who offered invaluable guidance and support during the project and suggestions that greatly improved this manuscript. We thank all TWG members and other stakeholders involved in data collation, service prioritisation and developing policy questions that guided our study.

## Disclosure of artificial intelligence (AI) use

 Not applicable.

## Ethical issues

 Ethical approval was not necessary as no primary or individual-level data were used.

## Conflicts of interest

 Authors declare that they have no conflicts of interest.

## Disclaimer

 The funder had no involvement in the research design, data collection, analysis, writing of this paper, or decision to submit. The views expressed in this paper are those of the authors and not those of the funder, nor of the authors’ respective organisations.

## Endnotes


^[1]^Total health spending was US$ 86 in 2022,^17^ equivalent to US$ 97 in 2023 following inflation adjustment using the World Bank Gross Domestic Product deflator.^35^ Around 20% of health spending is allocated to national-level programme and management activities,^44^ which are not included in our cost estimates. This results in spending of around US$ 77.6 to compare against. However, while the 181 prioritised services that account for our estimated US$ 74.41 per capita capture key service provision in Kyrgyzstan, they are not exhaustive of what national spending estimates capture and are therefore higher.

## Supplementary files


Supplementary file 1. Empty UHC Compendium Costing Model and Templates.

Supplementary file 2. Costing Tool Calculations Summary.

Supplementary file 3. Additional Assumption Tables and Timeline.

Supplementary file 4. Medicines and Populations in Need.

Supplementary file 5. List of 181 Prioritised Services With Utilisation and Cost Per Capita Estimates.
