# Supplementary material for: Costing Health Benefit Packages Using the WHO UHC Compendium: A Proof-of-Concept Study in Kyrgyzstan
Source: Int J Health Policy Manag. 2026 May 5;15:9333. doi: 10.34172/ijhpm.9333 (PMC13498982; doi:10.34172/ijhpm.9333)
Supplement: Supplementary file 1 [file ijhpm-15-9333-s002.pdf]

Article title: Costing Health Benefit Packages Using the WHO UHC Compendium: A Proof-of-Concept Study in Kyrgyzstan

Journal name: International Journal of Health Policy and Management (IJHPM)

Authors' information: Gerard Joseph Abou Jaoude[1]*¶, Charlie Nederpelt[2]¶, Saltanat Zhetibaeva[3], Gavin Surgey[2], Parisa Kamali Sadeghian[4], Neha Misra[4], Eliza Aidyldaeva[3], Mirlan Atakulov[5], Edil Orozaliev[5], Sven Neelsen[6], Christel Vermeersch[6], Baktygul Kambaralieva[7], Jolene Skordis[1], Tom Palmer[1], Rob Baltussen[2], Baktygul Isaeva[3], Hassan Haghparast-Bidgoli[1]

[1]Institute for Global Health, University College London, London, UK.

[2]Radboud University Medical Center, Nijmegen, The Netherlands.

[3]Ministry of Health, Bishkek, Kyrgyzstan.

[4]World Health Organization, Geneva, Switzerland.

[5]Mandatory Health Insurance Fund, Bishkek, Kyrgyzstan.

[6]World Bank, Washington, DC, USA.

[7]Japan International Cooperation Agency Kyrgyz Republic Office, Bishkek, Kyrgyzstan.

***Correspondence to:** Gerard Joseph Abou Jaoude; Email: gerard.jaoude.15@ucl.ac.uk

¶Both authors contributed equally to this paper.

Citation: Abou Jaoude GJ, Nederpelt C, Zhetibaeva S, et al. Costing health benefit packages using the WHO UHC compendium: a proof-of-concept study in Kyrgyzstan. Int J Health Policy Manag. 2026;15:9333. doi:10.34172/ijhpm.9333

Supplementary file 2. Costing Tool Calculations Summary

Calculations used to estimate costs per UHCC service bundle in the model are summarized below. Service bundle costs are summed across relevant platforms.

Notation

- Service delivery platforms: $p \in \{\text{COM, OPT, 1RL, 2RL}\}$
  - COM = Community-based
  - OPT = Outpatient/clinic
  - PRE = Pre-hospital level
  - 1RL = First referral level
  - 2RL = Second referral level
- Let:
  - U_p= Percentage Utilisation Distribution for platform p
  - N= Annual number of visits or inpatient days

- D= Duration of inpatient stay in days
- P_{need}= Percentage of population in need
- $C_{\text{visit},p}$= Cost per visit or inpatient day at platform p, based on the type of visit or inpatient stay
- W= Health worker wage per minute
- t_{proc}= Procedure duration in minutes
- s_{action}= Percentage share of action
- s_{HP}= Percentage share of health product
- C_{med}= Cost per medicine unit, based on route of administration and dosage form
- d_{dose}= Medicine doses per day
- T_{days}= Annual number of treatment days
- C_{IVD}= Cost per diagnostic/IVD unit, based on the type of diagnostic/IVD
- N_{IVD}= Annual number of diagnostic/IVD units required
- C_{Adj}= Cost per treatment adjunct unit, based on the type of treatment adjunct
- N_{Adj}= Annual number of treatment adjunct units required
- $u_{\text{cons},p}$= Uptick for consumables at platform p
- $u_{\text{over},p}$= Uptick for overheads at platform p
- C_{excl}= Costs excluding treatment adjuncts, consumables and overheads
- N_{pin}= Number of people in need
- U_{acc}= Current or target percentage of people in need accessing the service
- M= Multimorbidity factor equal to half the ratio between the pooled prevalence of chronic multimorbidity and morbidity in LMICs

1) Visit / Inpatient Stay Direct Labour Component

$$C_{\text{visit}} = \sum_{p} \left(P_{\text{need}} \times C_{\text{visit},p} \times N \text{ or } D \times U_p\right)$$

2) Procedures Direct Labour Component

$$C_{\text{proc}} = \sum_{p} \left(t_{\text{proc}} \times W \times s_{\text{action}} \times U_p\right)$$

3) Medicines

$$C_{\text{med}} = \sum_{p} \left(C_{\text{med}} \times s_{\text{action}} \times s_{\text{HP}} \times d_{\text{dose}} \times T_{\text{days}} \times U_p\right)$$

4) Diagnostics / IVD

$$C_{\text{IVD}} = \sum_{p} \left(C_{\text{IVD}} \times s_{\text{action}} \times N_{\text{IVD}} \times U_p\right)$$

5) Treatment Adjuncts

$$C_{\text{Adj}} = \sum_{p} \left(C_{\text{Adj}} \times s_{\text{action}} \times N_{\text{Adj}} \times U_p\right)$$

6) Consumables

$$C_{\text{cons}} = \sum_{p} \left(u_{\text{cons},p} \times C_{\text{excl}} \times U_p\right)$$

7) Overheads

$$C_{\text{over}} = \sum_{p} \left(u_{\text{over},p} \times C_{\text{excl}} \times U_p\right)$$

8) Total Service Cost per Service User

$$C_{\text{user}} = C_{\text{visit}} \times M + C_{\text{proc}} + C_{\text{med}} + C_{\text{IVD}} + C_{\text{Adj}} + C_{\text{cons}} + C_{\text{over}}$$

9) Total Service Cost

$$C_{\text{total}} = C_{\text{user}} \times N_{\text{pin}} \times U_{acc}$$
