# Supplementary material for: Costing Health Benefit Packages Using the WHO UHC Compendium: A Proof-of-Concept Study in Kyrgyzstan
Source: Int J Health Policy Manag. 2026 May 5;15:9333. doi: 10.34172/ijhpm.9333 (PMC13498982; doi:10.34172/ijhpm.9333)
Supplement: Supplementary file 2 [file ijhpm-15-9333-s003.pdf]

Article title: Costing Health Benefit Packages Using the WHO UHC Compendium: A Proof-of-Concept Study in Kyrgyzstan

Journal name: International Journal of Health Policy and Management (IJHPM)

Authors' information: Gerard Joseph Abou Jaoude[1]*¶, Charlie Nederpelt[2]¶, Saltanat Zhetibaeva[3], Gavin Surgey[2], Parisa Kamali Sadeghian[4], Neha Misra[4], Eliza Aidyldaeva[3], Mirlan Atakulov[5], Edil Orozaliev[5], Sven Neelsen[6], Christel Vermeersch[6], Baktygul Kambaralieva[7], Jolene Skordis[1], Tom Palmer[1], Rob Baltussen[2], Baktygul Isaeva[3], Hassan Haghparast-Bidgoli[1]

[1]Institute for Global Health, University College London, London, UK.

[2]Radboud University Medical Center, Nijmegen, The Netherlands.

[3]Ministry of Health, Bishkek, Kyrgyzstan.

[4]World Health Organization, Geneva, Switzerland.

[5]Mandatory Health Insurance Fund, Bishkek, Kyrgyzstan.

[6]World Bank, Washington, DC, USA.

[7]Japan International Cooperation Agency Kyrgyz Republic Office, Bishkek, Kyrgyzstan.

***Correspondence to:** Gerard Joseph Abou Jaoude; Email: gerard.jaoude.15@ucl.ac.uk

¶Both authors contributed equally to this paper.

Citation: Abou Jaoude GJ, Nederpelt C, Zhetibaeva S, et al. Costing health benefit packages using the WHO UHC compendium: a proof-of-concept study in Kyrgyzstan. Int J Health Policy Manag. 2026;15:9333. doi:10.34172/ijhpm.9333

Supplementary file 3. Additional Assumption Tables and Timeline

Table S1. Scaling factor assumptions used to estimate variations in reference costs by different types of outpatient visits or inpatient days

**Type of visit or inpatient stay**	**Scaling factor relative to reference cost**
Visit type	
Comprehensive visit	100% - Reference
Brief visit	75%
Preventive care visit	75%
Expanded visit	150%
Facility based visit for procedure / surgery	175%
Emergency unit visit	225%
Inpatient stay type	
Inpatient stay for condition / disease	100% - Reference
Inpatient stay for procedure / surgery	125%

Table S2. Laboratory and diagnostic unit costs (2023 USD)

**Type of diagnostic test**	**Cost per unit**
Point of care testing	$ 2.78
Basic laboratory tests	$ 2.78
Advanced laboratory tests	$ 14.91
Genomic testing	$ 203.28

Table S3. Uptick percentages used to estimate consumable/products and overheads

Feldsher - Family group practices	Family medicine centres	District hospital	Regional and tertiary hospitals
Product uptick			
3.67%	1.81%	4.84%	6.00%
Overhead uptick			
11.64%	11.96%	31.25%	24.12%

Figure S1. Key costing milestones and timeline with stakeholder involvement

Period	Milestones	Stakeholders involved
**September 2023**	1. Agreement on use of UHCC/SPDI and costing approach<br>2. SPDI training and introduction to key concepts in costing, planned tool development and costing approach	Stakeholders involved:<br>1. Policymakers and Technical Working Groups (TWGs)<br>2. TWG leads, assessment and costing sub-groups – facilitated by the international consultants
**October 2023**	1. Mapping of Kyrgyzstan health system in SPDI to reflect current and aspirational service delivery<br>2. Continued training and support on use of SPDI	Stakeholders involved :<br>1. TWGs<br>2. TWG leads, assessment and costing sub-groups – facilitated by the international consultants
**November 2023 – February 2024**	1. Finalised list of 424 services for consideration in priority setting deliberations<br>2. Agreement on costing methodology and staged approach, involving initial 'high-level' estimates followed by 'refined' estimates for prioritised services	Stakeholders involved:<br>1. Policymakers and TWGs<br>2. Policymakers and TWGs
**March – April 2024**	1. Evidence collation and 1.5-month review of 'high-level' cost analysis input data<br>2. Training and continuous support on use of costing templates	Stakeholders involved :<br>1. TWGs and international consultants<br>2. TWG leads, assessment and costing sub-groups – facilitated by the international consultants
**May – June 2024**	1. Finalised 'high-level' cost analysis<br>2. Prioritisation of 186 services, informed by 'high-level' cost analysis results and evidence on other decision criteria	Stakeholders involved :<br>1. International consultants<br>2. TWGs and policymakers
**October 2024 – January 2025**	1. Evidence collation and two-month review of 'refined' cost analysis input data<br>2. Finalised 'refined' cost analysis results for 186 services prioritised for inclusion in an updated benefits package	Stakeholders involved :<br>1. TWGs and international consultants<br>2. International consultants
