# Supplementary material for: Costing Health Benefit Packages Using the WHO UHC Compendium: A Proof-of-Concept Study in Kyrgyzstan
Source: Int J Health Policy Manag. 2026 May 5;15:9333. doi: 10.34172/ijhpm.9333 (PMC13498982; doi:10.34172/ijhpm.9333)
Supplement: Supplementary file 3 [file ijhpm-15-9333-s005.pdf]

Article title: Costing Health Benefit Packages Using the WHO UHC Compendium: A Proof-of-Concept Study in Kyrgyzstan

Journal name: International Journal of Health Policy and Management (IJHPM)

Authors' information: Gerard Joseph Abou Jaoude[1]*¶, Charlie Nederpelt[2]¶, Saltanat Zhetibaeva[3], Gavin Surgey[2], Parisa Kamali Sadeghian[4], Neha Misra[4], Eliza Aidyldaeva[3], Mirlan Atakulov[5], Edil Orozaliev[5], Sven Neelsen[6], Christel Vermeersch[6], Baktygul Kambaralieva[7], Jolene Skordis[1], Tom Palmer[1], Rob Baltussen[2], Baktygul Isaeva[3], Hassan Haghparast-Bidgoli[1]

[1]Institute for Global Health, University College London, London, UK.

[2]Radboud University Medical Center, Nijmegen, The Netherlands.

[3]Ministry of Health, Bishkek, Kyrgyzstan.

[4]World Health Organization, Geneva, Switzerland.

[5]Mandatory Health Insurance Fund, Bishkek, Kyrgyzstan.

[6]World Bank, Washington, DC, USA.

[7]Japan International Cooperation Agency Kyrgyz Republic Office, Bishkek, Kyrgyzstan.

***Correspondence to:** Gerard Joseph Abou Jaoude; Email: gerard.jaoude.15@ucl.ac.uk

¶Both authors contributed equally to this paper.

Citation: Abou Jaoude GJ, Nederpelt C, Zhetibaeva S, et al. Costing health benefit packages using the WHO UHC compendium: a proof-of-concept study in Kyrgyzstan. Int J Health Policy Manag. 2026;15:9333. doi:10.34172/ijhpm.9333

Supplementary file 5. List of 181 Prioritised Services With Utilisation and Cost Per Capita Estimates

Table S1. List of service names, percentage utilisation and estimated costs per capita based on existing utilisation (2023 USD)

Service name	Service utilisation	Cost per capita (existing service utilisation), 2023 USD
[additional] Additional diagnostics of anal cancer and management with systemic therapy and/or radiation therapy	25%	0.02
[core] Diagnosis of arrhythmias and management with medications	25%	0.05

[core] Diagnosis of acute asthma exacerbations and management with medications	25%	0.01
[core] Early diagnosis of breast cancer	25%	0.03
[core] Screening of breast cancer	25%	0.50
[additional] Management of breast cancer with radiation therapy	27%	0.01
[core] Assessment of cardiovascular risks	25%	0.77
[core] Diagnosis of stroke and longitudinal management with medications	25%	1.45
[additional] Additional management of acute stroke with i.v. medications	25%	0.24
[additional] Additional diagnostics for stroke	25%	0.04
[additional] Screening for cervical cancer	35%	0.03
[core] Assessment of behavioural disorders and management with medications and psychosocial interventions	11%	0.05
[core] Early detection of childhood cancer	25%	0.50
[core] Diagnosis of acute lymphoblastic leukemia and management with surgery, systemic therapy and/or radiation therapy	25%	0.01
[additional] Management of acute lymphoblastic leukemia with systemic therapy and stem cell transplantation	27%	0.00
[core] Diagnosis of burkitt lymphoma and management with surgery, systemic therapy and/or radiation therapy	25%	0.00
[core] Diagnosis of hodgkins lymphoma and management with systemic therapy and/or radiation therapy	25%	0.00
[core] Diagnosis of wilms tumor and management with surgery, systemic therapy and/or radiation therapy	25%	0.01
[additional] Diagnosis of wilms tumor	25%	0.00
[additional] Management of complications of wilms tumor treatment	25%	0.00
[core] Diagnosis of retinoblastoma and management with surgery, systemic therapy and/or radiation therapy	25%	0.00
[additional] Management of retinoblastoma with systemic therapy, laser therapy, stem cell transplantation and/or radiation therapy	27%	0.00
[core] Management of childhood cancer emergencies (general)	25%	0.01
[core] Diagnosis of COPD and longitudinal management with medications	25%	0.13
[core] Diagnosis of acute COPD exacerbations and management with medications	25%	0.01
[additional] Additional management of acute exacerbation of COPD	25%	0.00
[core] Prevention of chronic non-communicable diseases	25%	0.51
[core] Diagnosis of diabetes mellitus and management with counselling and medications	60%	4.04

[additional] Additional management of diabetes mellitus with home glucose monitoring	60%	0.02
[additional] Additional management of diabetes mellitus with home glucose monitoring and statins	60%	1.22
[core] Diagnosis of diabetic emergencies and management with medications	60%	0.82
[core] Diagnosis of diabetic neuropathy and management with footcare	60%	0.19
[additional] Diagnosis of diabetic retinopathy and longitudinal management with laser photocoagulation	60%	0.20
[additional] Additional diagnostics of ileus and intestinal obstruction and management with surgery	25%	0.00
[additional] Additional diagnostics of appendicitis and management with surgery	25%	0.00
[core] Diagnosis of acute kidney injury and management with medications	25%	0.01
[additional] Additional diagnostics of acute kidney injury and management with surgery	25%	0.01
[additional] Additional diagnostics of urinary retention or obstruction and management with surgery	25%	0.01
[core] Screening and initial diagnosis of eye disorders in children	25%	1.49
[core] Diagnosis of refractive error and management with spectacles in children	25%	0.79
[core] Diagnosis of amblyopia and strabismus and management of amblyopia with eye patching	25%	0.04
[core] Diagnosis of cataract and management with surgery in adults	25%	1.00
[additional] Additional management of cataract with surgery	59%	0.01
[core] Diagnosis of retinopathy of prematurity and management with laser therapy	25%	0.00
[core] Diagnosis of retinoblastoma and management with medications and surgery	59%	0.00
[core] Counselling and support for healthy nutrition	25%	0.87
[core] Diagnosis of heart failure and longitudinal management with medications	25%	0.04
[core] Diagnosis of acute heart failure and management with medications	25%	0.02
[additional] Additional management of heart failure with implantable cardioverter-defibrillator (ICD)	25%	0.00
[additional] Additional management of heart failure with non-invasive mechanical ventilation	25%	0.00
[core] Diagnosis of hypertension and management with medications	42%	1.71
[additional] Additional diagnostics for hypertension	42%	0.33

[core] Diagnosis of hypertensive emergencies and management with medications	42%	0.26
[core] Diagnosis of acute ischaemic heart disease and management with medications	25%	0.73
[core] Diagnosis of ischaemic heart disease and longitudinal management with medications	25%	0.01
[additional] Additional management of acute ischaemic heart disease with PCI	25%	0.00
[additional] Additional management of ischaemic heart disease with surgical interventions	25%	0.02
[core] Diagnosis of urticaria and management with medications	25%	0.04
[core] Diagnosis of iron deficiency anaemia and management with medication and supplements	25%	0.23
[additional] Additional diagnostics for iron deficiency anaemia	25%	0.04
[core] Assessment of psychosis and management with basic psychosocial support and medications	11%	0.10
[additional] Management of psychosis with psychological treatment	11%	0.03
[core] Assessment of bipolar disorder and management with basic psychosocial support and mood stabilizing medication	11%	0.25
[additional] Management of bipolar disorder with psychological treatment	11%	0.07
[core] Assessment of moderate-severe depression and management with basic psychosocial support, psychological treatment and oral antidepressants	11%	1.30
[core] Assessment and management of self-harm and suicide risk with basic psychosocial support and psychological treatment	11%	0.00
[core] Assessment of epilepsy and management with basic psychosocial support and medications	25%	0.49
[additional] Additional diagnostics of epilepsy and seizures	25%	0.00
[core] Assessment of dementia (including alzheimers disease) and management with psychosocial support and medications	25%	0.02
[additional] Additional management of testicular torsionwith surgery	25%	0.01
[core] Screeening of oral cancer	25%	0.05
[core] Screening and prevention of oral diseases and other conditions (including dental caries)	25%	0.16
[core] Assessment of overweight and obesity and management with behavioural and/or psychological therapy and surgery	25%	3.02
[core] Assessment of physical activity and management with counselling and behavioural support	25%	0.56
[core] Prevention of primary immunodeficiency disorders	25%	0.28

[core] Management of primary immunodeficiencies with surgical intervention	25%	0.00
[additional] Additional diagnostics for primary immunodeficiencies	25%	0.00
[additional] Additional management for complications related to primary immunodeficiencies	25%	0.00
[core] Prevention of rheumatic heart disease with medications	25%	0.00
[core] Diagnosis of sleep disorders and management with education	25%	0.00
[core] Assessment opioid overdose and management with medications	4%	0.11
[core] Assesment of opioid withdrawal syndome and management with medications and psychosocial support	4%	0.01
[core] Assesment of drug withdrawal syndome (cannabis, psychostimulants and others) and management with medications and psychosocial support	4%	0.02
[additional] Additional management of inflammatory arthritis with surgical procedures	25%	0.01
[core] Screening and diagnosis of thyroid disorders	25%	0.08
[core] Prevention of poor growth	25%	0.06
[core] Diagnosis of wasting and management with counselling and medications	25%	0.05
[core] Diagnosis of micronutrient deficiencies and management with counselling and medications	25%	0.19
[core] Diagnosis of meningitis and management with medications	60%	0.15
[additional] Additional management of other CNS infections	60%	0.02
[core] Diagnosis of ear infections and management with medications	60%	0.17
[additional] Additional management of ear infection complications with surgical procedure	60%	0.11
[core] Diagnosis of other ear, nose and throat (ENT) infections and management with medications	60%	2.74
[additional] Additional diagnostics for other ear, nose and throat (ENT) infections	60%	0.54
[core] Diagnosis of enteric infections and management with oral, intramuscular and intravenous therapies	36%	0.83
[core] Prevention of viral hepatitis with counselling, vaccination and harm reduction services	60%	0.30
[additional] Additional prevention of viral hepatitis with vaccination	60%	0.19
[core] Diagnosis of chronic hepatitis C and management with medications	16%	1.08
[core]Diagnosis of chronic hepatitis B and management with oral antivirals	16%	0.77
[core] Diagnosis of chronic hepatitis B and management with medications	16%	0.08

[additional] Additional diagnosis of chronic hepatitis	16%	0.06
[core] Prevention of HIV through counselling and provision of condoms and lubricants	53%	0.53
[core] Oral PrEP and dapivirine vaginal ring	78%	1.31
[core] Post-exposure prophylaxis (PEP)	78%	0.61
[core] Prevention and diagnosis of lower respiratory infections and management with antimicrobials at community level (for children under 5)	60%	0.01
[core] Prevention and diagnosis of lower respiratory infections and management with antimicrobials and oxygen in a facility	60%	0.77
[additional] Advanced management of lower respiratory infections with additional diagnostics and therapeutics	60%	0.23
[core] Prevention of measles through behavioural change	95%	0.00
[core] Diagnosis of measles and management with medications in the treatment unit	60%	0.04
[core] Prevention of rabies - Post Exposure Prophylaxis	60%	0.00
[core] Diagnosis of rabies and management with medications and palliative care	60%	0.00
[core] Management of necrotising soft tissue infections with surgical intervention	60%	0.01
[core] Diagnosis of helminthiasis and management with medication	60%	0.48
[core] Prevention of STIs with counselling	60%	0.36
[core] Post-exposure prophylaxis (PEP) for STIs	60%	0.06
[core] Syndromic diagnosis of STIs and reproductive tract infections and management with medications	29%	0.06
[core] Diagnosis of STIs and reproductive tract infections with laboratory tests and management with medications	29%	0.13
[additional] Additional diagnostics for STIs and reproductive tract infections	60%	0.19
[core] Diagnosis of prostatitis and management with medications	60%	0.05
[core] Screening for tuberculosis initiated by provider	7%	0.00
[core] Prevention of tuberculosis with oral medication	7%	0.22
[core] Case finding of tuberculosis initiated by patient	53%	0.49
[core] Diagnosis of tuberculosis initiated by patient	7%	0.23
[core] Management of drug-sensitive tuberculosis with oral medications	53%	0.55
[core] Diagnosis and management of drug-resistant tuberculosis with medications	53%	0.92
[additional] Additional diagnostics for tuberculosis	53%	0.01
[additional] Management of tuberculosis with surgery	53%	0.01
[core] Syndromic diagnosis of UTIs and management with oral medications	60%	0.39

[core] Routine immunizations for children	90%	3.04
[core] Recommendations for children receiving vaccinations from immunization programmes with certain characteristics	90%	0.89
[core] Recommended vaccinations for adults in immunization programmes with certain characteristics	90%	0.71
[core] Hepatitis B vaccination for individuals in special high risk population groups	90%	0.09
[core] Covid vaccination	90%	2.11
[core] Recognition and initial response to interpersonal violence	25%	0.18
[core] Recognition and initial response to child maltreatment and youth violence	25%	1.30
[additional]Additional management of coagulation disorder with medications	25%	0.00
[core] Counselling and prevention of bites and envenoming injuries	25%	0.00
[core] Basic emergency care for bites and envenoming injuries	25%	0.02
[core] Advanced emergency care for bites and envenoming injuries	25%	0.05
[core] HIV testing services and treatment	78%	1.31
[core] Recognition and initial response to intimate partner violence	25%	0.21
[core] Recognition and initial response to sexual violence	25%	0.01
[core] Counselling and prevention of mechanical injuries	25%	0.00
[core] Basic emergency care of acute mechanical injury	25%	0.32
[core] Advanced emergency care of acute mechanical injury	25%	0.37
[additional] Haemodialysis in a second referral hospital	25%	0.00
[additional] Surgical procedures for mechanical injury in a second referral hospital	25%	0.00
[additional] Advanced prehospital emergency care setting	25%	0.04
[core] Counselling and prevention of poisoning, toxic and environmental exposures	25%	0.03
[core] Basic emergency care for environmental exposures (including drowning)	25%	0.03
[core] Basic emergency care for poisoning and toxic exposures	25%	0.05
[additional] Intravenous medication in an outpatient setting for poisoning and toxic exposures	25%	0.01
[core] Advanced emergency care for poisoning and toxic exposures	25%	0.01
(core) Basic emergency care for thermal and chemical injuries (burns)	25%	0.23
(core) Advanced emergency care for thermal and chemical injuries	25%	0.40
[additional] Mechanical ventilation for thermal and chemical injuries (burns) in first referral hospital	25%	0.01
[core] Identification and response of violence against older people	25%	0.01

[core] Diagnosis of pregnancy and management of abortion with systemic therapy	42%	0.35
[additional] Management of abortion with oral medication	42%	0.03
[core] Diagnosis of pregnancy and management of abortion with surgery	42%	0.08
[core] Diagnosis of complications related to abortion and management with systemic therapy and surgery	42%	0.90
[core] Routine care during pregnancy	94%	5.56
[core] Management of antenatal complications	60%	3.71
[core] Diagnosis of congenital critical heart defects and management with systemic therapy and surgery	60%	0.58
[core] Diagnosis of congenital neural tube defects and management with...	60%	0.00
[core] Diagnosis of congenital hypothyroidism and management with...	60%	0.06
[core] Diagnosis of congenital talipes and management with...	60%	0.02
[core] Diagnosis of cleft lip and/or palate and management with...	60%	0.03
[core] Diagnosis of ectopic pregnancy and management with medication and surgery	60%	0.29
[additional] Additional management of ectopic pregnancy with surgery	60%	0.05
[core] Support for healthy growth and development	60%	0.61
[core] Wellness assessments during childhood	60%	0.30
[core] Wellness assessments during adulthood	60%	0.31
[core] Routine care during labour and childbirth	100%	1.69
[core] Management of complications during labour and childbirth	99%	2.64
[core] Promotion of menstrual health	60%	0.16
[core] Diagnosis of painful menstrual bleeding and management with medications and supportive care	60%	0.05
[core] Diagnosis of heavy menstrual bleeding and management with medications and supportive care	60%	0.36
[core] Routine care for postpartum women and newborn after first hour of birth	96%	0.50
[core] Diagnosis and management of neonatal complications after 24 hours of delivery	91%	2.15
[core] Screening, testing and counselling on family planning and choice of contraception	65%	2.33

NOTE: Costs per capita do not always reflect 'complete' services and are based on health actions selected. For example, some advanced technologies, medications and procedures were removed or deemed not to be feasible when adapting services to the country context. In turn, for some services it was only deemed feasible to cover a subset of a population-in-need at certain facility-levels. Costs per capita therefore reflect relevant actions selected within services and/or subsets of populations-in-need that were costed.
